# Prevalence of iodine deficiency among pregnant women in Gwembe and Sinazongwe districts of Southern Province, Zambia: a cross-sectional study

**DOI:** 10.1186/s40795-020-00397-w

**Published:** 2020-12-09

**Authors:** Trevor Kaile, Bornwell Sikateyo, Masauso M. Phiri, Charles Michelo

**Affiliations:** 1 Department of Pathology and Microbiology, The University of Zambia, Ridgeway Campus, Lusaka, Zambia; 2 Department of Medical Education Development, The University of Zambia, School of Medicine, Ridgeway Campus, Lusaka, Zambia; 3 The University of Zambia, School of Public Health, Ridgeway Campus, Lusaka, Zambia

**Keywords:** Pregnant women, Iodine, Urine iodine, Iodine deficiency, Goitre, Iodization of salt

## Abstract

**Background:**

Maternal iodine deficiency is one of the common causes of morbidity and mortality during pregnancy. Maternal iodine deficiency during pregnancy is associated with a number of adverse outcomes such as abortion, stillbirth, congenital anomalies, perinatal mortality and irreversible mental retardation. A study conducted in Zambia among pregnant women in 2013 on the prevalence of iodine deficiency showed that iodine deficiency was not a public health concern. The previous study used Urine Iodine concentration (UIC) as a marker of iodine deficiency among the pregnant women. Our study was conducted to assess the prevalence of iodine deficiency among pregnant women in Gwembe and Sinazongwe districts of Southern Province, Zambia, using urine iodine concentration and goitre presence by manual palpation.

**Methods:**

We carried out a community based, cross sectional study in rural areas of Gwembe and Sinazongwe districts between April 2016 to March 2018. Data were collected from 412 pregnant women by a multistage cluster sampling technique. The presence of a goitre was examined by manual palpation and urinary iodine concentration was determined by the Ultra Violet Method using PerkinElmer Labda UV Spectrometer equipment made in Jena Germany (Model 107,745). As part of the existing baseline data, we used results of a 2013 countrywide study (*n* = 489) for household salt iodine content which showed a greater than 40 ppm at 76.2%, between 15 and 40 ppm at 19.21% and less than 15 ppm at 4.59%. Statistical analysis was done using Stata version 14.0. The outputs of analysis are presented as median and Interquartile range (IQR) as the urine data were not normally distributed. Further, the categorical and independent variables were presented as proportions (percentages) to describe the distribution and trends in the target sample population.

**Results:**

The median Urine Iodine concentration (UIC) of the pregnant women was 150 μg/L (Interquartile Range (IQR): 100–200 μg/L). Based on the UIC, There were 49% pregnant women who had inadequate iodine intake with urine iodine concentration of less than 150 μg/L, 34.0% had UIC of 150–249 μg/L indicating adequate iodine intake, 13.0% with UIC of 250–499 μg/L indicating more than adequate iodine intake, and 5.0% with UIC of above 500 μg/L indicating excessive iodine intake. To determine whether the women had access to iodized salt, we used baseline data from 2013 Zambia national survey for iodine concentration in household salt samples as being an average of 40 ppm, which also showed that 95.41% households consumed adequately iodized salt (≥15 ppm). The prevalence of goitre in our study was very low at 0.02% among the pregnant women of all ages who participated in the study (18–49 years).

**Conclusion:**

Iodine deficiency was still not a public health concern among the pregnant women of Gwembe and Sinazongwe districts of Southern Province in Zambia. Goitre prevalence has remained very low in this study area. The UIC and goitre observations were consistent with the Zambia National Food and Nutrition Commission findings in 2013 report. However, our study showed more pregnant women with insufficient than adequate iodine status indicating the risk of developing IDD is still high in this region. It also reinforces the argument that strengthening of the existing salt iodization program is needed in order to make a homogenous iodated salt available to the communities. The National Food and Nutrition Commission of Zambia needs to find innovative ways of sensitizing people about the adverse effects of IDDs and how these could be prevented. It is recommended that iodine supplementation be introduced as part of the package of Antenatal clinic care for all pregnant women.

## Background

Iodine is an essential micronutrient used by the body to synthesize thyroid hormones that are necessary for normal growth, development and metabolism [1–3]. Low iodine intake results in inadequate production of thyroid hormones that results in a syndrome known as iodine deficiency disorders (IDD) [4].

Iodine deficiency (ID) is a global health problem regardless of the level of development of the country [4, 5]. It was estimated in 2011 that 1.8 billion people consumed inadequate iodine and were at risk of developing IDD [6]. The most vulnerable from the consequences of ID were pregnant women and their new born babies. The results of iodine deficiency are abortion, still birth, mental retardation of children born from such mothers, low birth weight, perinatal death, stunted infant growth, neuromotor, intellectual, behavioural and cognitive impairments, neonatal hypothyroidism and goitre [7–14]. WHO stated that the most prevalent cause of brain damage but yet preventable disorder was iodine deficiency [15, 16]. There is paucity of data on the prevalence of iodine deficiency in pregnancy in Zambia.

Iodine Deficiency Disorders had been a great public health concern in Zambia leading to a number of prevalence studies [17–22]. A goitre prevalence of 38% was reported among children in Serenje District in Central Province as far back as 1951 [18]. This was followed by a nationwide comprehensive survey on IDD that involved 37 Districts from 8 provinces using goitre detected by neck palpation as a maker of iodine deficiency in 1971. The study found a goitre prevalence of 50.5% with a range from 27 to 81% across the whole country [18]. In 1989, a survey involving, 1163 children aged between 3 and 14 years in Livingstone in the Southern Province recorded a goitre prevalence of 18% [19]. In 1991, another survey observed a goitre prevalence of 68% in 105 pupils aged between 8 and 12 years in Choma in Southern Zambia [19]. A United Nations Children’s Fund (UNICEF) survey involving 2505 pupils drawn from all the nine provinces of Zambia recorded a goitre prevalence ranging from 9 to 82% with a mean of 31.6% across the country [20]. A 2011 IDD impact survey showed a marked stride in improving adequate iodine status in the population with an IDD prevalence of 14% using mid urine iodine concentration [21].

The 2013 survey conducted among pregnant women showed that ID was not a public health concern [14]. However, no study has been conducted to simultaneously assess prevalence of ID among pregnant women in rural Zambia after reinforcement of monitoring of iodized salt imported into Zambia 1998. Gwembe and Sinazongwe districts of Southern Province in Zambia were among the high prevalence areas for Iodine deficiency in surveys done before 2011.

Therefore, this study was conducted to assess the prevalence of Iodine deficiency in pregnant women in Gwembe and Sinazongwe districts of Southern Province in Zambia as a way of determining the effectiveness of iodine intake among the pregnant women in Zambian population.

## Methods

### Study design

This was a community based, cross-sectional, quantitative study with both descriptive and analytic elements.

### Study setting

The study was conducted between April 2016 to March 2018 in 6 and 12 Census Supervisory Areas (CSA) of Gwembe and Sinazongwe respectively. A Census Supervisory Areas is a lowest census unit in Zambia containing at least 80–120 households in a rural area and 120–150 households in an urban area with an average of 6 family members per household.

### Sample size

A single proportion sample size calculation formula was used to determine optimal sample size for estimating the prevalence of iodine deficiency. A sample size of 412 pregnant women was computed based on an estimated 32% prevalence of goitre in 1998 in the same area. In 2011, the nationwide survey showed 26.4% prevalence of UIC <  150 μg/L [15], at 95% confidence level, 5% margin of error, design effect of 1.5 and non response rate of 10%. In this cross sectional study, our response rate was a 100%.

### Sampling technique

A convenient sampling was used to select the pregnant women from the rural health centre catchment areas on first-come-first-recruitment basis using the census supervisory areas (CSA). The women were recruited as they came for their antenatal assessments. The pregnant women were identified as they came to the clinic by Rural Health centre staff (nurses, midwives and Medical Assistants). Individual participants were then selected using a systematic approach.

### Data collection method

In the surveys, data were collected using a pre-tested structured questionnaire. Socio-demographic data were collected using questions adapted from the Demographic and Health Surveys (DHS) questionnaire [19]. The questionnaire was prepared in English and translated into local language (Chitonga). It was administered in either/both languages according to participant preference. Data were collected by trained Rural Health Centre staff (nurses, Midwives, Medical Assistants and clinical Laboratory staff).

### Goitre examination

Presence of goitre was examined by trained and experienced clinic staff using manual palpation techniques and graded according to the criteria of WHO/UNICEF/ICCIDD [2]. The goitre was then classified as, grade 0 (no goitre), 1 (Palpable but not visible goitre) and grade 2 (palpable and visible goitre).

### Urine sample collection and laboratory analysis

Random urine samples were collected from every 10th pregnant woman in disposable plastic cup specimen containers and transferred into screw capped plastic vials. Vials were sealed with parafilm to prevent leakage and evaporation. They were transported to Lusaka, University of Zambia, School of Medicine Laboratory where the urine samples were frozen and stored at − 20^0^ C until they were analysed at the Food and Drugs laboratory in Lusaka, Zambia. Urine Iodine Concentration (UIC) was measured in duplicate by Ultra Violet Method using PerkinElmer Labda UV Spectrometer equipment, Jena, Germany. The UIC was categorized according to WHO/UNICEF/ICCIDD recommended epidemiological criteria for assessing iodine intake in pregnant women. Accordingly, the UIC was categorized as less than 150 μg/L, 150–249 μg/L, 250–499 μg/L, and equal and greater than 500 μg/L. [2]

### Data analysis

The data was analysed using Strata SE v.40. Histograms and the Kolmogorov-Simmov test were used to check for normality of numeric data before further analysis. Descriptive analysis was done using median and interquartile range (IQR) and percentage as appropriate for categorical and continuous variables. UIC was skewed and not normally distributed. Non-parametric Mann-Whitney U test and Wallis test were used to compare the median UIC and salt iodine concentration and differences between groups of categorical independent variables.

Bivariate and multivariate logistic regression analyses were used to identify the variables associated with goitre status. Independent variables were selected and included in the analysis to observe their effects on the development of goitre. In multivariate logistic regression analyses was undertaken to identify risk and predisposing factors to the development of goitre. These factors were; maternal education, and awareness of iodized salt and IDD, maternal age, parity, gestational age and salt iodine level.

## Results

### Study participants characteristics

This is shown in Tables 1 and 2 as Population distribution and characteristics of study participants in Rural Gwembe and Sinazongwe districts, Southern Province, Zambia, April 2016 – March 2018. They were almost four times more participants from Sinazongwe than Gwembe districts. All the 412 pregnant women were recruited as participants in their third trimester. The median age of the participants was 21.1 years but above 18 years of age. The legal age of consent for study participation is 18 years in Zambia. This could explain why there were no women below the age of 18 years participating in the study. The majority of the pregnant women were primagravidae (63%), Gestational age at recruitment was third trimester for 412 women (100%). The median number of pregnancies was 1. Of these women, 10% were illiterate, 49% had primary education, 41% had secondary education. Prevalence of goitre (0.02%), which was very low, was not associated with age, parity, lack of basic education, employment status, consumption of sorghum or sources of water.

**Table 1:** Site population and age distribution data of the study participants

**Site**	**Number of Respondents**	
Gwembe	153 (23.2%)	
Sinazongwe	479 (75.8%)	
Total	632 (100%)	
	**Age distribution (in years)**	
**Age**	**Gwembe**	**Sinazongwe**
Less than 20	84 (59%)	270 (56%)
20–25	35 (24%)	138 (29%)
26–30	7 (5%)	32 (7%)
31–35	9 (6%)	23 (5%)
36–40	7 (5%)	12 (3%)
More than 40	1 (0.7%)	4 (1%)
**Totals**	143	479

**Table 2:** Study population education levels and parity

	**Educational profiles**	
**Education**	**Gwembe**	**Sinazongwe**
None	31 (22%)	42 (9%)
Primary	71 (50%)	234 (49%)
Junior High School	35 (24%)	131 (27%)
Senior High School	6 (4%)	66 (14%)
Totals	143	479
	**Parity**	
**Parity**	**Gwembe**	**Sinazongwe**
More than 1	24 (17%)	78 (16%)
1	20 (14%)	104 (22%)
0	99 (69%)	297 (62%)
Totals	143	479

The pregnant women in our study were mainly subsistence farmers (64%) who grow only enough food for their families. They consumed mostly maize (92%), the regional staple food, although a few grew and consumed sorghum as well.

The water sources that were available to these participants were safe water from either borehole or tap water (77%). By contrast, only about 23% used the alternative sources from rivers, streams and wells. The average family size was six (+/− 2).

### Occupation status, food and water sources

The majority of the women were farmers who grew enough of their own food, mainly maize, to feed their families. The main source of water for their daily use was borehole water which did not have iodine added to it.

### Knowledge of iodised salt and iodine deficiency disorders and practice of use of iodized salt

More than 80% of the participants indicated that they had heard about iodized salt through radio campaigns (NFNC). Other sources of information were Community Health workers (CHW). Iodized salt usage in our study was 96% of the women iodized coarse salt and 1.6% fine iodised salt.

### Desk review on iodine concentration of household salt samples (*n* = 489, 2013)

The review data of iodine concentrations (ppm) in the salt samples from households ranged from less than 15 to above 40 ppm. Adequately iodated salt with 15–40 ppm iodine was 19.21%, iodine content less than 15 ppm was 4.59% and above 40 ppm was 76.2% [14].

The UIC was tabulated according to the WHO /UNICEF/ICCIDD criteria. The table showed the median UIC in third trimester as 150 μg/L (IQR: 100–200 μg/L). There were 49% pregnant women who had inadequate iodine intake with urine iodine concentration of less than 150 μg/L, 34.0% had UIC of 150–249 μg/L indicating adequate iodine intake, 13.0% with UIC of 250–499 μg/L indicating more than adequate iodine intake, and 5.0% with UIC of above 500 μg/L indicating excessive iodine intake,.

### Prevalence of goitre

**Fig. 1:**
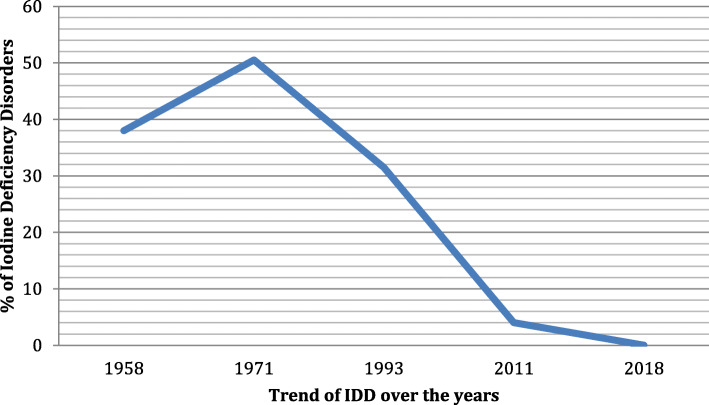
The prevalence of iodine deficiency over the years

**Fig. 2:**
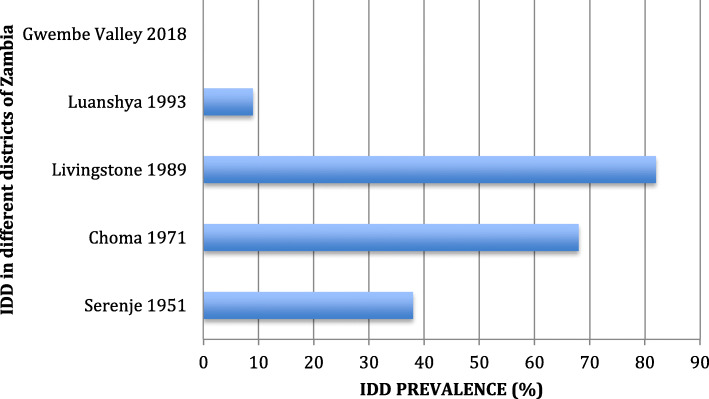
The IDD prevalence in different districts of Zambia since the start of IDD monitoring

Figures 1 and 2 showed the trend of goitre prevalence over time in the different towns and districts in Zambia. It is shown that the prevalence of goitre among the pregnant women in Gwembe and Sinazongwe districts study area was 0.02% (only 1 woman out of 412 had a palpable goitre) this was grade 1 goitre. There was no visible grade 2 goitre among the pregnant women who participated in the study. The trend shows that there was a significant reduction of goitre in the population from 1951 to 2018.

## Discussion

The study findings concurs with the observations of 2011 and 2013 countrywide survey that reported that Iodine deficiency was no longer a public health concern in Zambia. This was confirmed by use of both clinical signs for the presence of goitre in pregnant women as well as biochemical methods of determining random urine iodine concentration. The goitre prevalence was very low at 0.02% and Urinary Iodine concentration median at 150 μg/L, with an interquartile range of 100–200 μg/L. The urinary iodine concentrations represents recent iodine intake and could be used as a good indicator of iodine status [23]. It has been observed that prevalence of goitre tended to increase in women of multiparity [14, 21, 22, 24], who were of older age group, although age by itself did not have much effect except for women who had repeated pregnancies [24]. This trend was not observed in our study as the majorityThe majority of the pregnant women were primagravidaes and below the age of 25 years (77%) as a result could be less at risk of goitre (Tables 1 and 2).

Studies carried out in Denmark, France and Turkey all showed that illiteracy among pregnant women and guardians was associated with predisposition to Iodine deficiency in pregnant women and school going age [25–30]. The explanation given is that was that educated women with disposable income tended to afford iodated salt and that they had enough knowledge on the importance of consumption of iodated salt. However, multivariate analysis of our data did not show any link between goitre development and maternal literacy levels (Tables 1 and 2), this was demonstrated by the very low goitre prevalence observed in the study communities (Figs. 1 and 2) with a significant down ward trend since goitre monitoring started in Zambia.

In Table 3, the iodine Urine concentrated were grouped according to the WHO/UNICEF/ICCIDD criteria. We observed a median UIC of 150 μg/L in 34% of the pregnant women. This was within the range of 150–249 μg/L which was indicative of iodine sufficiency or adequate iodine intake in line with WHO/UNICEF/ICCIDD criteria [2]. In this study the median UIC was 150 μg/L, which was within the recommended adequate minimum median UIC for pregnant women. However, in Table 3, 49% of our study participants had insufficient recommended UIC (< 150 μg/L) which was below the cut off for adequate iodine intake. This is almost half of the study participants showing iodine intake lower than the recommended minimum median UIC for pregnant women which suggests that half of the pregnant women and their new born may still suffer from adverse effects of iodine deficiency in Gwembe and Sinazongwe districts.

**Table 3:** Urinary iodine concentration levels in pregnant women in their third trimester in Gwembe and Sinazongwe of Southern Province, Zambia from April 2016 to March 2018 (*n* = 218)

Iodine intake status	Urine Iodine concentration (μg/L)	Number (n)	Percent (%)
Insufficient	< 150	106	48.6
Adequate	150–249	74	34.0
More than Adequate	250–499	28	12.8
Excessive	≥ 500	10	4.6
Total		218	100

Our study results are in contrast to the previous study carried out to determine the iodine status of pregnant women in Zambia of 2011. This study reported a prevalence of Iodine deficiency with UIC less 150 μg/L at 26.4% [14] and a median UIC of 264 μg/L which was above the normal range of 150–249 μg/L. [14] This study also reported a normal median UIC of 150 μg/L but which was negatively skewed. We observed a reduction on the median UIC in the current study suggesting a relaxation on monitoring of consumed iodated salt in the communities. This suggests that the pregnant women were still at risk of IDD thus predisposing their new borns also to IDD.

The WHO/UNICEF/ICCIDD criteria for prevalence of goitre greater than 5% indicated a public health problem [2]. The prevalence of goitre in the current study was 0.02% which was far much lower than the cut off where IDD would be considered a public health problem. The prevalence was far lower in these areas and suggests a consistent downward trend of the goitre through out the country as shown in (Figs. 1 and 2). The overall reduction in whole country may be an indication that the iodine supplementation strategies put in place by the National Food and Nutrition Commission of the Republic of Zambia are yielding results and a continuous monitoring of imported salts for human and animal consumption should be strengthened (NFNC). However with an increase in insufficient iodine intake status and a reduction in iodine status for adequate iodine intake by the pregnant women shown in Table 3, the IDD problem may still resurface in Zambia. This finding is significant and we propose that the National Food and Nutrition Commission needs to reinforce monitoring and compliance of salt iodization and other iodine fortification and supplementation in other food staffs. The observed increase in iodine concentration in salt samples used by households may help in mitigating increased iodine intake [27].

The pregnant women in our study were mainly farmers (64%) who grew enough food for their families. Further, these families grew and consumed mostly maize (92%), the regional staple food, although a few grew and consumed sorghum as well. Maize consumption did not predispose to iodine deficiency. This observation suggested good nutrition status for the study population.

The water sources that were available to these participants were safe water from either borehole or tap water (77%). By contrast, only about 23% used the alternative sources from rivers, streams and wells. Sources of water were not associated with goitre or inadequate iodine intake.

It is now universally accepted that universal salt iodization (USI) consisting of iodizing all salt meant for human and animal consumption is the most affordable way of alleviating IDD [2]. The National Food and Nutrition Commission of Zambia of 2013 found 19.21% of household salt sample to be using adequately iodated salt (15–40 ppm iodine), 4.59% (at less than 15 ppm) and 76.2% (at above 40 ppm). This showed that most salt manufacturers were complying with iodization of salt process, although most them were over iodizing the salt [27]. This observation of high iodine in salt may be desirable considering that almost half (49%) of the pregnant women had less that 150 μg/L of median UIC meaning that they were not taking enough iodine in their daily consumption hence higher iodine levels in household salt was a better strategy of increasing iodine status in pregnant women. This study findings reinforces the suggestion that poor adherence to salt regulation by salt producers requires continuous effective monitoring system at ports of entry for all imported and locally produced salt in Zambia. The improvement of effective coverage of adequately iodated salt by manufacturers through effective monitoring would assist in mitigating insufficient and excessive iodine intake among pregnant women in Zambia. The low iodine status among women suggests that there is a lack of knowledge about IDD and consumption of iodised salt among pregnant women in Gwembe and Sinazongwe districts implying that dissemination of IDD and its effects on the foetus has not been conveyed comprehensively at consumer community level.

To the best of our knowledge, this is the second study in Zambia to assess the prevalence of goitre among pregnant women using clinical and biochemical methods. This study had some limitations in that a single random sample of urine was collected for analysis of iodine. Urinary iodine tended to have a daily variation depending on individual iodine intake of each woman. Therefore urinary iodine concentration could only be used to assess the iodine status of a population but not of individuals [23]. Micronutrients such as selenium that was known to enhance iodine deficiency were not measured in our study. The other limitation was that we depended on baseline data from National Food and Nutrition Commission of Zambia for household iodine content in salt. We did not measure iodine content of water and other food staffs. Inter individual variations may have occurred among the clinical staff when palpations for goitre were done. Ultrasound detection of goitre may be more sensitive but may not be practicable in low resource settings like Zambia.

## Conclusion

Iodine deficiency was still not a public health concern among the pregnant women of Gwembe and Sinazongwe districts of Southern Province in Zambia. Goitre prevalence has remained very low in this study area. The UIC and goitre observations were in line with the Zambia National Food and Nutrition commission findings of 2013 report. However, our study showed more pregnant women with insufficient than adequate iodine status indicating a risk to developing IDD is still high in this region. It also reinforces the argument that strengthening of the existing salt iodization program is needed in order to make a homogenous iodated salt available to the communities. The National Food and Nutrition Commission of Zambia needed to find innovative ways of sensitizing people about the adverse effects of IDDs and how these could be prevented. It is recommended that iodine supplementation be introduced as part of the package of Antenatal clinic care for all pregnant women.

## Data Availability

Data and materials availability for this manuscript can be requested from the corresponding author in reasonable time.

## Abbreviations

NHDS: National Health Demographic survey
FAO: Food and Agricultural Organization
ICCIDD: International Council on Control of Iodine Deficiency Disorders
IDD: Iodine Deficiency Disorders
UIC: Urinary Iodine Concentration
USI: Universal salt iodization
WHO: World Health Organization
